# Relationship between platelet aggregation and stroke risk after percutaneous coronary intervention: a PENDULUM analysis

**DOI:** 10.1007/s00380-021-02003-w

**Published:** 2022-01-01

**Authors:** Yuji Matsumaru, Takanari Kitazono, Kazushige Kadota, Koichi Nakao, Yoshihisa Nakagawa, Junya Shite, Hiroyoshi Yokoi, Ken Kozuma, Kengo Tanabe, Takashi Akasaka, Toshiro Shinke, Takafumi Ueno, Atsushi Hirayama, Shiro Uemura, Takeshi Kuroda, Atsushi Takita, Atsushi Harada, Raisuke Iijima, Yoshitaka Murakami, Shigeru Saito, Masato Nakamura

**Affiliations:** 1 Division of Stroke Prevention and Treatment, Department of Neurosurgery, Faculty of Medicine, University of Tsukuba, 1-1-1 Tennodai, Tsukuba, Ibaraki 305-8575 Japan; 2 Department of Clinical Medicine, National University Corporation Kyushu University, Fukuoka, Japan; 3 Department of Cardiology, Kurashiki Central Hospital, Okayama, Japan; 4 Division of Cardiology, Saiseikai Kumamoto Hospital Cardiovascular Center, Kumamoto, Japan; 5 Department of Cardiovascular Medicine, Shiga University of Medical Science, Shiga, Japan; 6 Division of Cardiology, Osaka Saiseikai Nakatsu Hospital, Osaka, Japan; 7 Cardiovascular Medicine Center, Fukuoka Sanno Hospital, Fukuoka, Japan; 8 Division of Cardiology, Department of Internal Medicine, Teikyo University, Tokyo, Japan; 9 Division of Cardiology, Mitsui Memorial Hospital, Tokyo, Japan; 10 Department of Cardiovascular Medicine, Wakayama Medical University, Wakayama, Japan; 11 Division of Cardiology, Department of Medicine, Showa University School of Medicine, Tokyo, Japan; 12 Department of Cardiovascular Medicine, Fukuoka Kinen Hospital, Fukuoka, Japan; 13 Department of Cardiology, Osaka Police Hospital, Osaka, Japan; 14 Department of Cardiology, Kawasaki Medical School, Okayama, Japan; 15 Medical Science Department, Daiichi Sankyo Co., Ltd., Tokyo, Japan; 16 Data Intelligence Department, Daiichi Sankyo Co., Ltd., Tokyo, Japan; 17 Medical Information Department, Daiichi Sankyo Co., Ltd., Tokyo, Japan; 18 Division of Cardiovascular Medicine, Toho University Ohashi Medical Center, Tokyo, Japan; 19 Department of Medical Statistics, School of Medicine, Toho University, Tokyo, Japan; 20 Division of Cardiology and Catheterization Laboratories, Shonan Kamakura General Hospital, Kanagawa, Japan

**Keywords:** Ischemic stroke, Platelet aggregation, Stents, Thrombosis

## Abstract

In patients undergoing percutaneous coronary intervention (PCI) with a stent, high on-treatment platelet reactivity may be associated with an increased risk of stroke. This post hoc analysis of the PENDULUM registry compared the risk of post-PCI stroke according to on-treatment P2Y_12_ reaction unit (PRU) values. Patients aged ≥ 20 years who underwent PCI were stratified by baseline PRU (at 12 and 48 h post-PCI) as either high (HPR, > 208), optimal (OPR, > 85 to ≤ 208), or low on-treatment platelet reactivity (LPR, ≤ 85). The incidences of non-fatal ischemic and non-ischemic stroke through to 12 months post-PCI were recorded. Almost all enrolled patients (6102/6267 [97.4%]) had a risk factor for ischemic stroke, and most were receiving dual antiplatelet therapy. Of the 5906 patients with PRU data (HPR, *n* = 2227; OPR, *n* = 3002; LPR, *n* = 677), 47 had a non-fatal stroke post-PCI (cumulative incidence: 0.68%, ischemic; 0.18%, non-ischemic stroke). Patients with a non-fatal ischemic stroke event had statistically significantly higher post-PCI PRU values versus those without an event (*P* = 0.037). The incidence of non-fatal non-ischemic stroke was not related to PRU value. When the patients were stratified by PRU ≤ 153 versus > 153 at 12–48 h post-PCI, a significant difference was observed in the cumulative incidence of non-fatal stroke at 12 months (*P* = 0.044). We found that patients with ischemic stroke tended to have higher PRU values at 12–48 h after PCI versus those without ischemic stroke.

Clinical trial registration: UMIN000020332.

**Supplementary Information:**

The online version contains supplementary material available at 10.1007/s00380-021-02003-w.

## Introduction

Worldwide, stroke is one of the leading causes of disability and mortality [1] and is a major healthcare issue across Asia [2]. Data reported in 2013 showed that the incidence rates of stroke were 422/100,000 person-years [PY] for men and 212/100,000 PY for women in Japan, and the most common type of stroke in Japanese individuals is reported to be ischemic stroke (75.4%) [2].

Current Japanese guidelines state that antiplatelet agents, such as aspirin or clopidogrel, should be used for the secondary prevention of ischemic stroke [3]. Until recently, monotherapy was the established regimen [4]. However, the CHANCE trial, which compared 21 days of dual antiplatelet therapy (DAPT; clopidogrel plus aspirin) with aspirin alone in patients with mild stroke or transient ischemic attack within 24 h of onset, showed a significant benefit of DAPT in preventing recurrent stroke at 3 months without increasing bleeding events [5]. In contrast, the POINT trial compared 90 days of DAPT (clopidogrel plus aspirin) with aspirin alone and found a similar significant reduction in myocardial infarction (MI) and cardiovascular death at 3 months with DAPT, but there was a significant increase in major bleeding [6]. Based on this evidence, the Japanese guidelines recommended DAPT for 3 weeks after a stroke [3].

For some patients diagnosed with a stroke, response to antiplatelet treatment is poor [7], resulting in a recent investigative focus on the phenomenon of high on-treatment platelet reactivity (HTPR). For patients with HTPR, there is a negative impact on their clinical course, worsened prognosis, and an increased risk of recurrent vascular events [7, 8]. However, there is a paucity of definitive data on the topic since many reports are based on a small number of cases. Several researchers have used meta-analysis methodology to pool available information. The findings indicate that HTPR occurs in up to 65% of patients receiving antiplatelet monotherapy and 35% of those receiving DAPT [9–11], and that it doubles the risk of stroke/transient ischemic attack [10, 11].

Antiplatelet agents are prescribed for secondary prevention of thrombotic events, including cardiovascular death, ischemic stroke, MI, and stent thrombosis, in patients who have undergone percutaneous coronary intervention (PCI) with a stent [12, 13]. In the prospective ADAPT-DES registry including 11 US and European hospitals, data collected from 8665 patients who were prescribed aspirin and clopidogrel following placement of a drug-eluting stent (DES) indicated that HTPR on clopidogrel was related to stent thrombosis (*P* = 0.01) and MI (*P* = 0.001), and HTPR on clopidogrel and aspirin was inversely related to bleeding (*P* = 0.002 and *P* = 0.04, respectively) [14].

However, there are no reports of large-scale studies in East Asian populations that have measured P2Y_12_ reaction unit (PRU) values and examined the association with stroke. This is particularly important as East Asian individuals have a high bleeding risk and a low thrombotic risk compared with other races and ethnicities [15, 16]. The PENDULUM (Platelet rEactivity in PatieNts with DrUg eLUting stent and balancing risk of bleeding and IscheMic event) registry is a prospective, multicenter observational study of > 6000 Japanese patients who have undergone PCI with DES; in the published analysis of 1-year data, high PRU values (> 208) measured at 12–48 h post‐PCI were reported to be associated with cardiovascular events, but no association was found with hemorrhagic events [17].

The current post hoc analysis of data from the PENDULUM registry was conducted specifically to compare the risk of stroke after PCI according to PRU values.

## Materials and methods

### Study design and patients

Full details of the prospective, multicenter PENDULUM registry have been published [17]. In brief, patients who underwent PCI were enrolled between December 2015 and June 2017 from 67 medical institutions across Japan. In principle, all eligible patients were registered; the key eligibility criteria were age ≥ 20 years, an indication for PCI with DES, and administration of antiplatelet drugs. Treatment (drug type, dosage, and duration) was at the discretion of the attending physician. DAPT was based on the standard of care in Japan at the time the study was conducted (aspirin, 100 mg once daily [QD], increasing to 300 mg QD; clopidogrel, 300 mg loading dose, followed by 75 mg QD; prasugrel, 20 mg loading dose, followed by 3.75 mg QD). All patients provided written informed consent before study participation.

For the current analysis, patients were stratified by baseline PRU value [18] into three categories: high on-treatment platelet reactivity (HPR; PRU > 208), optimal on-treatment platelet reactivity (OPR; PRU > 85 to ≤ 208), and low on-treatment platelet reactivity (LPR; PRU ≤ 85). The VerifyNow^®^ system (Instrumentation Laboratory, Bedford, MA, USA) was used to measure platelet reactivity, and results were reported in PRU. Mandatory measurements were performed between 12 and 48 h post-index PCI, with subsequent optional measurements collected whenever possible.

The study was performed per the principles of the Declaration of Helsinki, and the Ethical Guidelines for Medical and Health Research Involving Human Subjects and was registered in the University hospital Medical Information Network (UMIN) Clinical Trial Registry (UMIN000020332). The study protocol and related documents were approved by the Ethics Committee at Toho University Ohashi Medical Center on 14 December 2015 (reference code: 15-71).

### Outcomes

Outcomes evaluated in this post hoc analysis were the incidences of non-fatal ischemic stroke and non-fatal non-ischemic stroke in each patient subgroup. Stroke was classified into ischemic stroke and non-ischemic stroke (hemorrhagic stroke, i.e., cerebral hemorrhage and subarachnoid hemorrhage). Non-fatal stroke was defined as a new neurological sign or symptom with a responsible lesion confirmed by computed tomography (CT) or magnetic resonance imaging (MRI). Ischemic stroke was defined as a new neurological sign or symptom with a new associated infarct confirmed by CT or MRI examination, regardless of whether neurological signs or symptoms persisted for more than 24 h.

### Statistical analysis

Kaplan–Meier analysis was done to show the incidences of events through to 12 months after the index PCI. For individuals who had multiple events of the same outcome, only the first event was counted. Patients who discontinued the study and those alive at the end of the observation period were censored. Univariate Cox regression models were used to estimate the hazard ratios (HR) and the 95% confidence intervals (CI), with OPR data used as reference. Summary statistics for PRU values at 12–48 h post-PCI were calculated for patients with or without each event; *P* values were calculated using *t*-test. Receiver-operating characteristic (ROC) analysis was performed to assess the association between PRU and post-PCI events. Statistical analyses were conducted using SAS version 9.4 (SAS Institute, Inc., Cary, NC, USA). All statistical tests were two-sided with a 5% level of significance.

## Results

### Patients

In total, 6422 patients were registered in the PENDULUM registry, of whom 6267 were included in the full analysis set. Among the evaluable patients, 2278/6267 (36.3%) were using beta-blockers. Common comorbidities included hypertension (5186/6267 [82.8%]), hyperlipidemia (4919/6267 [78.5%]), chronic kidney disease (estimated glomerular filtration rate [eGFR] ≤ 60 mL/min/1.73 m^2^; 2691/6267 [42.9%]), and diabetes mellitus (2767/6267 [44.2%]). In addition, there were 655/6267 (10.5%) patients with a history of ischemic stroke and 124/6267 (2.0%) with a history of cerebral hemorrhage [17].

5906 patients had PRU data available and were included in the present evaluation. Background details according to PRU (HPR, *n* = 2227; OPR, *n* = 3002; and LPR, *n* = 677) have been reported [17]. In brief, HPR patients had a mean age of 71.8 years, 989/2227 (44.4%) were aged ≥ 75 years, and 1650/2227 (74.1%) were male. Respective characteristics of OPR patients (69.1 years; 979/3002 [32.6%] ≥ 75 years; and 2451/3002 [81.6%] male) and LPR patients (68.5 years; 227/677 [33.5%]; and 528/677 [78.0%]) were generally similar.

### Outcomes

**Fig. 1:**
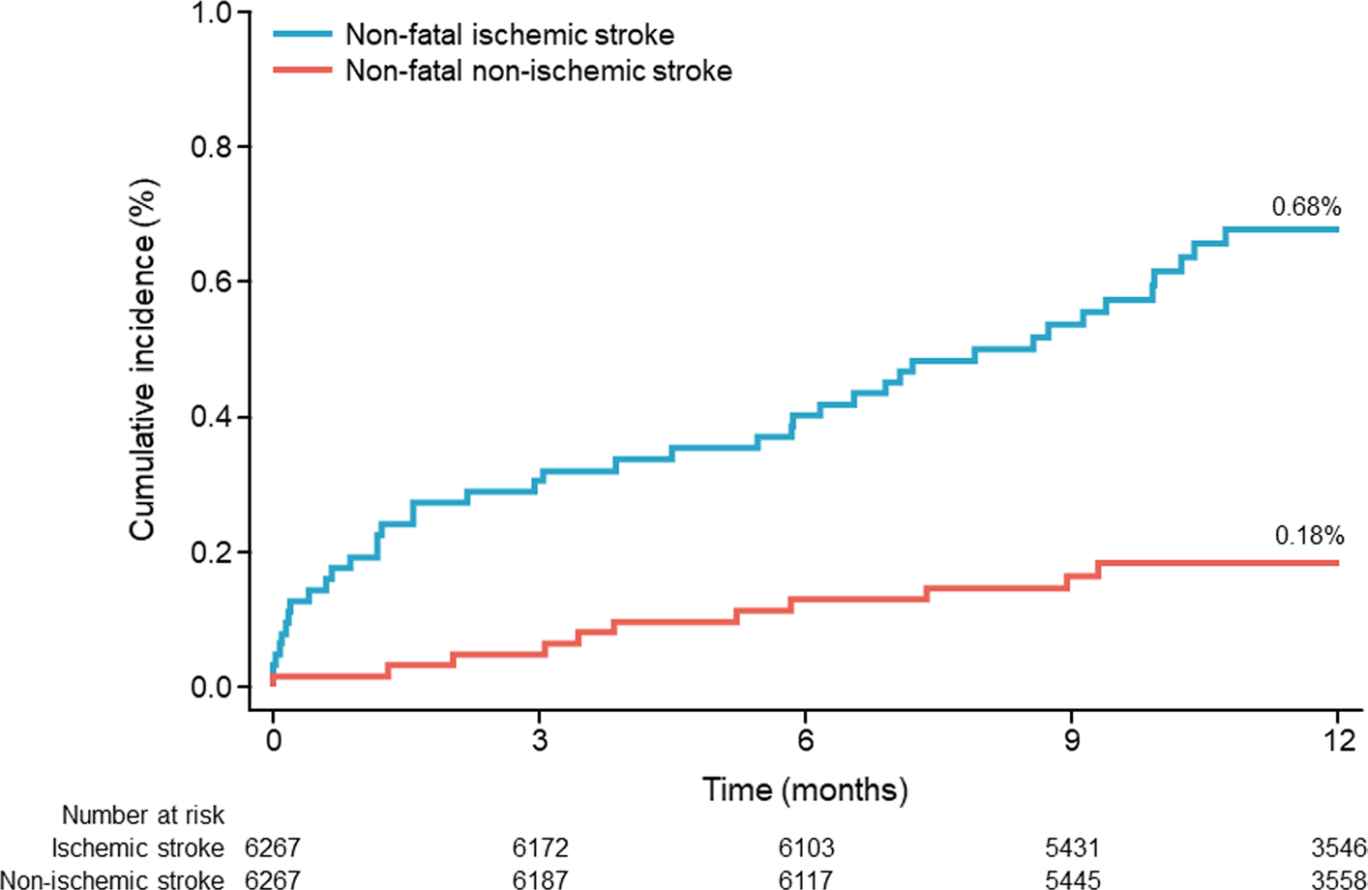
Cumulative incidence of stroke from 0 to 12 months after percutaneous coronary intervention

Overall, 51/6267 patients had a non-fatal stroke within 1 year after PCI. Of these, 40 patients had a non-fatal ischemic stroke (cumulative incidence 0.68%; 95% CI, 0.50–0.93) and 11 patients had a non-fatal non-ischemic stroke (cumulative incidence 0.18%; 95% CI, 0.10–0.33) (Fig. 1). In 5906 patients with available PRU, 37 patients had a non-fatal ischemic stroke (cumulative incidence 0.67%; 95% CI, 0.48–0.92) and 10 patients had a non-fatal non-ischemic stroke (cumulative incidence 0.18%; 95% CI, 0.10–0.33).

**Fig. 2:**
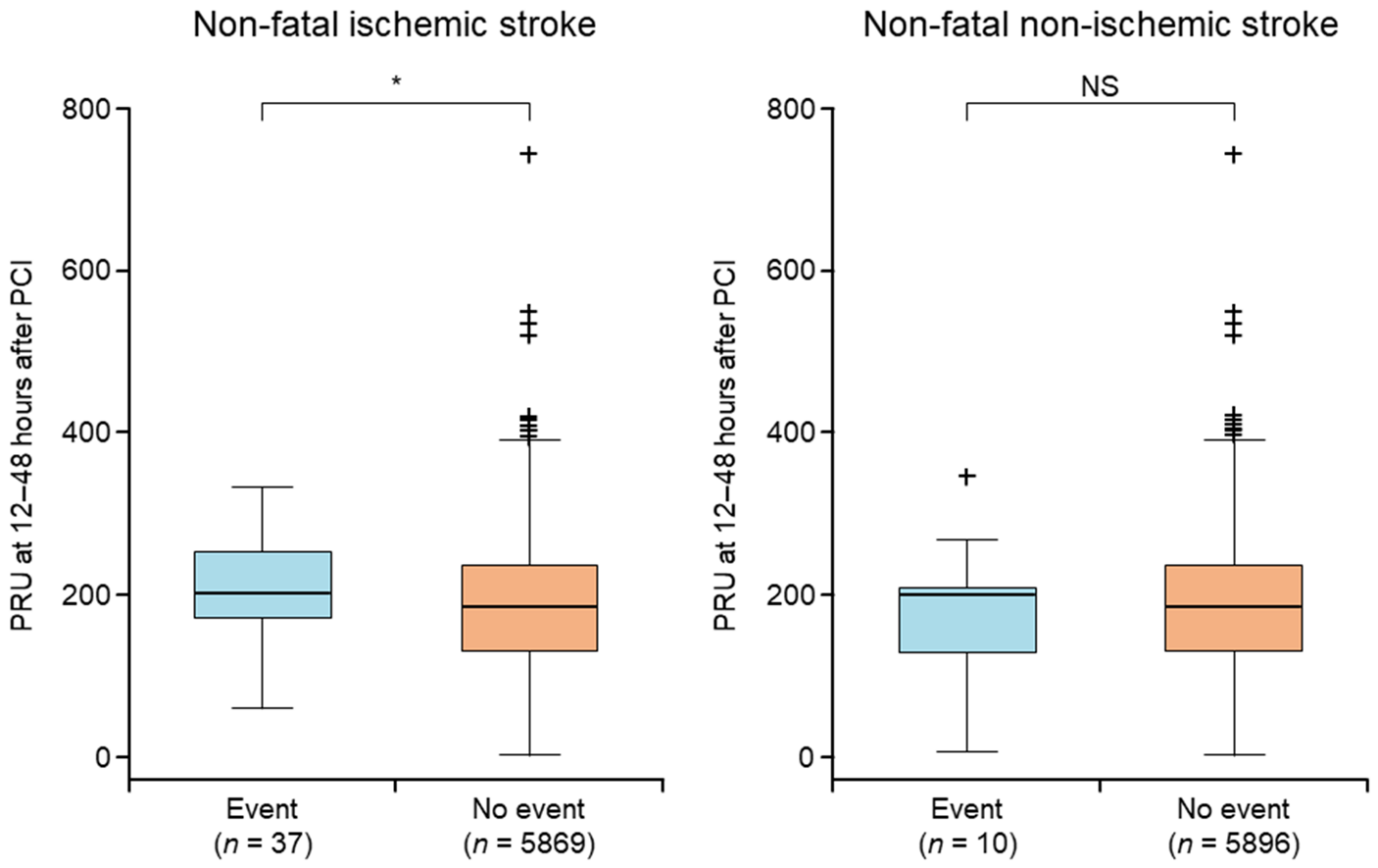
P2Y_12_ reaction units at 12–48 h after percutaneous coronary intervention according to the incidence of non-fatal ischemic stroke and non-fatal non-ischemic stroke. The box shows the 1st quartile, the 2nd quartile (median), and the 3rd quartile. The upper and lower bars show the measured values farthest from the median within 1.5 IQR from the box. Outliers of more than 1.5 IQR from the box are denoted by +. *IQR* interquartile range, *NS* not significant*, PCI* percutaneous coronary intervention, *PRU* P2Y_12_ reaction unit. *P < 0.05

PRU values in patients with and without non-fatal stroke events are shown in Table 1 and Fig. 2. Patients with an event of non-fatal ischemic stroke had statistically significantly higher post-PCI PRU values than patients without an event (*P* = 0.037). Conversely, there was no significant difference in PRU between patients with a non-fatal non-ischemic stroke event and those without an event.

**Table 1:** P2Y_12_ reaction units at 12–48 h after percutaneous coronary intervention in patients who did or did not suffer a non-fatal stroke event

P2Y_12_ reaction units	Non-fatal ischemic stroke		*P* value
	Yes (*n* = 37)	No (*n* = 5869)	
Mean (SD)	208.5 (67.0)	182.0 (77.1)	0.037
HPR,* n* (%)	17 (45.9)	2210 (37.7)	
OPR,* n* (%)	18 (48.6)	2984 (50.8)	
LPR,* n* (%)	2 (5.4)	675 (11.5)	

	Non-fatal non-ischemic stroke		
	Yes (*n* = 10)	No (*n* = 5896)	
Mean (SD)	178.4 (95.7)	182.2 (77.1)	0.878
HPR,* n* (%)	3 (30.0)	2224 (37.7)	
OPR,* n* (%)	5 (50.0)	2997 (50.8)	
LPR,* n* (%)	2 (20.0)	675 (11.4)	

*HPR* high P2Y_12_ reaction unit value, *LPR* low P2Y_12_ reaction unit value, *OPR* optimal P2Y_12_ reaction unit value, *SD* standard deviation

**Fig. 3:**
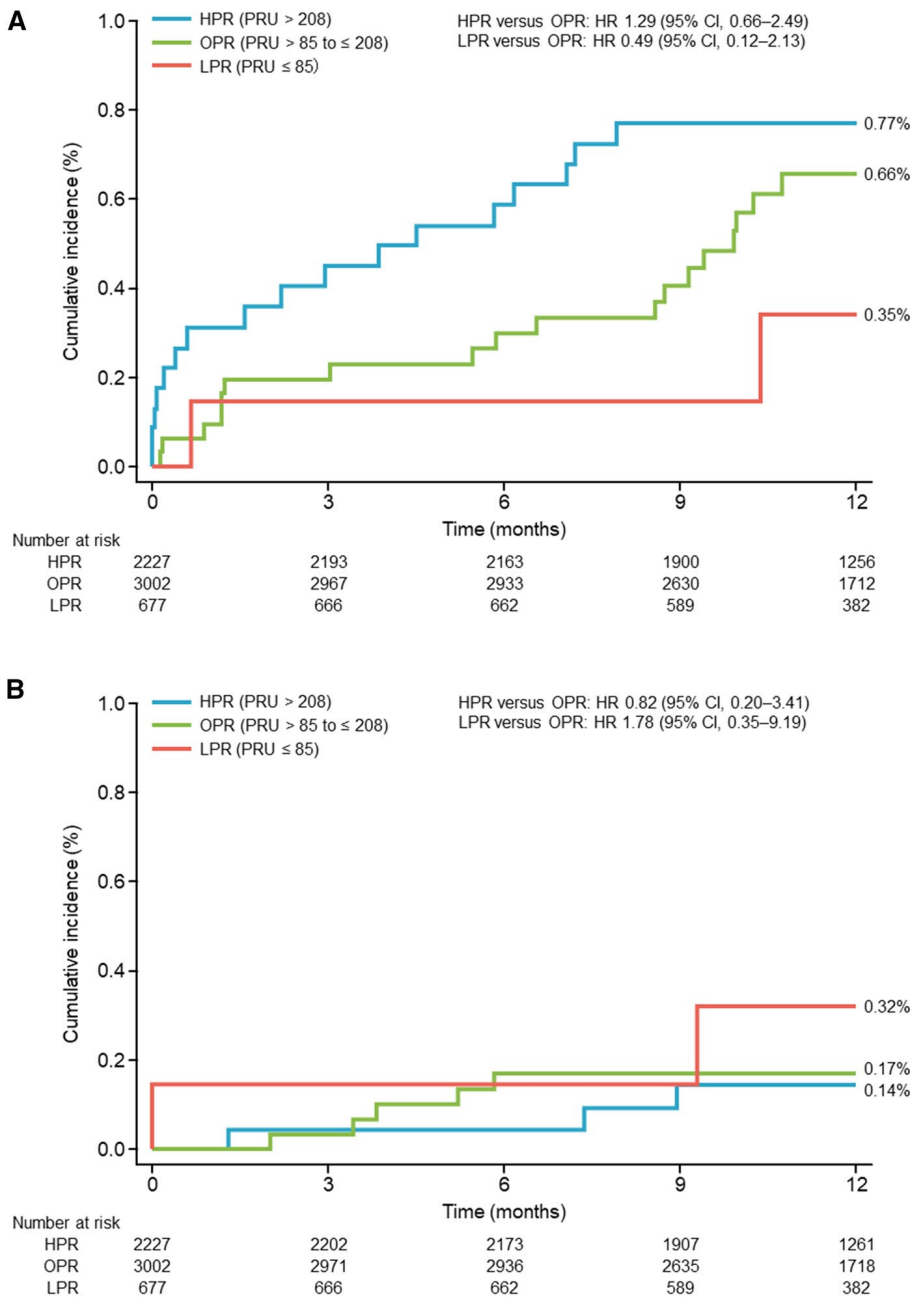
Incidence of stroke according to P2Y_12_ reaction unit value. **A** Non-fatal ischemic stroke. **B** Non-fatal non-ischemic stroke. *CI* confidence interval, *HPR* high P2Y_12_ reaction unit value, *HR* hazard ratio, *LPR* low P2Y_12_ reaction unit value, *OPR* optimal P2Y_12_ reaction unit value, *PRU* P2Y_12_ reaction unit

The cumulative incidence of non-fatal ischemic stroke by PRU category is shown in Fig. 3a. The cumulative incidence of events tended to increase as the PRU level increased, but the differences were not statistically significant. The incidence of non-fatal non-ischemic stroke was not related to PRU value (Fig. 3b).

The incidence of stroke (all events and by stroke type) according to patient characteristics are shown in Table 2. The atherothrombotic and lacunar stroke incidences increased with higher PRU values, but similar tendencies were not observed for cardiogenic stroke. Almost all patients (6102/6267 [97.4%]) had a risk factor for ischemic stroke (i.e., history of ischemic stroke, diabetes, hyperlipidemia, chronic kidney disease, or hypertension). Of the 40 patients with non-fatal ischemic stroke, 36 (90.0%) patients received DAPT at the time of the event.

**Table 2:** Incidence of stroke (overall and by subtype) according to patient characteristics

	*N*	All	Ischemic stroke						Non-ischemic stroke
			All	Non-cardiogenic			Cardiogenic	Other	
				All	Atherothrombotic	Lacunar			
All	6267	51 (0.8)	40 (0.6)	20 (0.3)	12 (0.2)	8 (0.1)	7 (0.1)	13 (0.2)	11 (0.2)
Age, years									
≥ 75	2324	23 (1.0)	20 (0.9)	12 (0.5)	7 (0.3)	5 (0.2)	2 (0.1)	6 (0.3)	3 (0.1)
< 75	3943	28 (0.7)	20 (0.5)	8 (0.2)	5 (0.1)	3 (0.1)	5 (0.1)	7 (0.2)	8 (0.2)
Sex									
Male	4909	42 (0.9)	31 (0.6)	15 (0.3)	8 (0.2)	7 (0.1)	6 (0.1)	10 (0.2)	11 (0.2)
Female	1358	9 (0.7)	9 (0.7)	5 (0.4)	4 (0.3)	1 (0.1)	1 (0.1)	3 (0.2)	0
Body weight, kg									
≤ 50	794	9 (1.1)	9 (1.1)	5 (0.6)	4 (0.5)	1 (0.1)	0	4 (0.5)	0
> 50	5326	41 (0.8)	30 (0.6)	14 (0.3)	7 (0.1)	7 (0.1)	7 (0.1)	9 (0.2)	11 (0.2)
Smoking									
Yes	1674	13 (0.8)	9 (0.5)	5 (0.3)	3 (0.2)	2 (0.1)	0	4 (0.2)	4 (0.2)
No	3951	29 (0.7)	22 (0.6)	10 (0.3)	6 (0.2)	4 (0.1)	6 (0.2)	6 (0.2)	7 (0.2)
Hypertension									
Yes	5186	44 (0.8)	36 (0.7)	19 (0.4)	11 (0.2)	8 (0.2)	7 (0.1)	10 (0.2)	8 (0.2)
No	1081	7 (0.6)	4 (0.4)	1 (0.1)	1 (0.1)	0	0	3 (0.3)	3 (0.3)
Hyperlipidemia									
Yes	4919	42 (0.9)	34 (0.7)	17 (0.3)	10 (0.2)	7 (0.1)	6 (0.1)	11 (0.2)	8 (0.2)
No	1348	9 (0.7)	6 (0.4)	3 (0.2)	2 (0.1)	1 (0.1)	1 (0.1)	2 (0.1)	3 (0.2)
Diabetes mellitus									
Yes	2767	23 (0.8)	18 (0.7)	8 (0.3)	5 (0.2)	3 (0.1)	4 (0.1)	6 (0.2)	5 (0.2)
No	3500	28 (0.8)	22 (0.6)	12 (0.3)	7 (0.2)	5 (0.1)	3 (0.1)	7 (0.2)	6 (0.2)
eGFR, mL/min/1.73 m^2^									
< 30	598	6 (1.0)	6 (1.0)	3 (0.5)	1 (0.2)	2 (0.3)	2 (0.3)	1 (0.2)	0
≥ 30 to < 60	2093	16 (0.8)	12 (0.6)	6 (0.3)	3 (0.1)	3 (0.1)	0	6 (0.3)	4 (0.2)
≥ 60	3431	27 (0.8)	20 (0.6)	9 (0.3)	6 (0.2)	3 (0.1)	5 (0.1)	6 (0.2)	7 (0.2)
Anemia, hemoglobin g/dL									
< 11	727	8 (1.1)	7 (1.0)	4 (0.6)	1 (0.1)	3 (0.4)	0	3 (0.4)	1 (0.1)
≥ 11 to < 13 (male) or ≥ 11 to < 12 (female)	1414	13 (0.9)	10 (0.7)	5 (0.4)	4 (0.3)	1 (0.1)	2 (0.1)	3 (0.2)	3 (0.2)
≥ 13 (male) or ≥ 12 (female)	3946	27 (0.7)	20 (0.5)	8 (0.2)	5 (0.1)	3 (0.1)	5 (0.1)	7 (0.2)	7 (0.2)
PRU value at 12–48 h after index PCI									
HPR (> 208)	2227	20 (0.9)	17 (0.8)	9 (0.4)	6 (0.3)	3 (0.1)	2 (0.1)	6 (0.3)	3 (0.1)
OPR (> 85 to ≤ 208)	3002	23 (0.8)	18 (0.6)	9 (0.3)	4 (0.1)	5 (0.2)	4 (0.1)	5 (0.2)	5 (0.2)
LPR (≤ 85)	677	4 (0.6)	2 (0.3)	1 (0.1)	1 (0.1)	0	1 (0.1)	0	2 (0.3)
*Cardiovascular status*									
ACS									
Yes	2015	22 (1.1)	19 (0.9)	9 (0.4)	6 (0.3)	3 (0.1)	3 (0.1)	7 (0.3)	3 (0.1)
No	4252	29 (0.7)	21 (0.5)	11 (0.3)	6 (0.1)	5 (0.1)	4 (0.1)	6 (0.1)	8 (0.2)
PAD									
Yes	421	3 (0.7)	3 (0.7)	1 (0.2)	1 (0.2)	0	0	2 (0.5)	0
No	5846	48 (0.8)	37 (0.6)	19 (0.3)	11 (0.2)	8 (0.1)	7 (0.1)	11 (0.2)	11 (0.2)
Heart failure									
Yes	850	9 (1.1)	8 (0.9)	4 (0.5)	2 (0.2)	2 (0.2)	0	4 (0.5)	1 (0.1)
No	5417	42 (0.8)	32 (0.6)	16 (0.3)	10 (0.2)	6 (0.1)	7 (0.1)	9 (0.2)	10 (0.2)
AF									
Yes	538	10 (1.9)	8 (1.5)	5 (0.9)	3 (0.6)	2 (0.4)	2 (0.4)	1 (0.2)	2 (0.4)
No	5729	41 (0.7)	32 (0.6)	15 (0.3)	9 (0.2)	6 (0.1)	5 (0.1)	12 (0.2)	9 (0.2)
*Medical history*									
MI									
Yes	1575	11 (0.7)	8 (0.5)	6 (0.4)	3 (0.2)	3 (0.2)	0	2 (0.1)	3 (0.2)
No	4661	40 (0.9)	32 (0.7)	14 (0.3)	9 (0.2)	5 (0.1)	7 (0.2)	11 (0.2)	8 (0.2)
Stroke									
Yes	741	11 (1.5)	9 (1.2)	5 (0.7)	3 (0.4)	2 (0.3)	0	4 (0.5)	2 (0.3)
No	5308	35 (0.7)	28 (0.5)	13 (0.2)	7 (0.1)	6 (0.1)	7 (0.1)	8 (0.2)	7 (0.1)
Hemorrhagic stroke									
Yes	124	1 (0.8)	0	0	0	0	0	0	1 (0.8)
No	5892	44 (0.7)	36 (0.6)	17 (0.3)	10 (0.2)	7 (0.1)	7 (0.1)	12 (0.2)	8 (0.1)
Ischemic stroke									
Yes	655	11 (1.7)	9 (1.4)	5 (0.8)	3 (0.5)	2 (0.3)	0	4 (0.6)	2 (0.3)
No	5396	35 (0.6)	28 (0.5)	13 (0.2)	7 (0.1)	6 (0.1)	7 (0.1)	8 (0.1)	7 (0.1)
Risk factor for ischemic stroke^a^									
Yes	6102	51 (0.8)	40 (0.7)	20 (0.3)	12 (0.2)	8 (0.1)	7 (0.1)	13 (0.2)	11 (0.2)
No	165	0	0	0	0	0	0	0	0
OAC at discharge									
Yes	610	8 (1.3)	5 (0.8)	1 (0.2)	0	1 (0.2)	2 (0.3)	2 (0.3)	3 (0.5)
No	5657	43 (0.8)	35 (0.6)	19 (0.3)	12 (0.2)	7 (0.1)	5 (0.1)	11 (0.2)	8 (0.1)

Data are *N* or* n* (%)

*ACS* acute coronary syndrome, *AF* atrial fibrillation, *eGFR* estimated glomerular filtration rate, *HPR* high P2Y_12_ reaction unit value, *LPR* low P2Y_12_ reaction unit value, *MI* myocardial infarction, *NSAID* non-steroidal anti-inflammatory drug, *OAC* oral anticoagulant, *OPR* optimal P2Y_12_ reaction unit value, *PAD* peripheral artery disease, *PRU* P2Y_12_ reaction unit

^a^History of ischemic stroke, diabetes, hyperlipidemia, eGFR < 60 mL/min/1.73 m^2^, or hypertension

The status of antiplatelet therapy at the time of the first event is shown in Online Resource 1. Most patients were receiving DAPT.

**Fig. 4:**
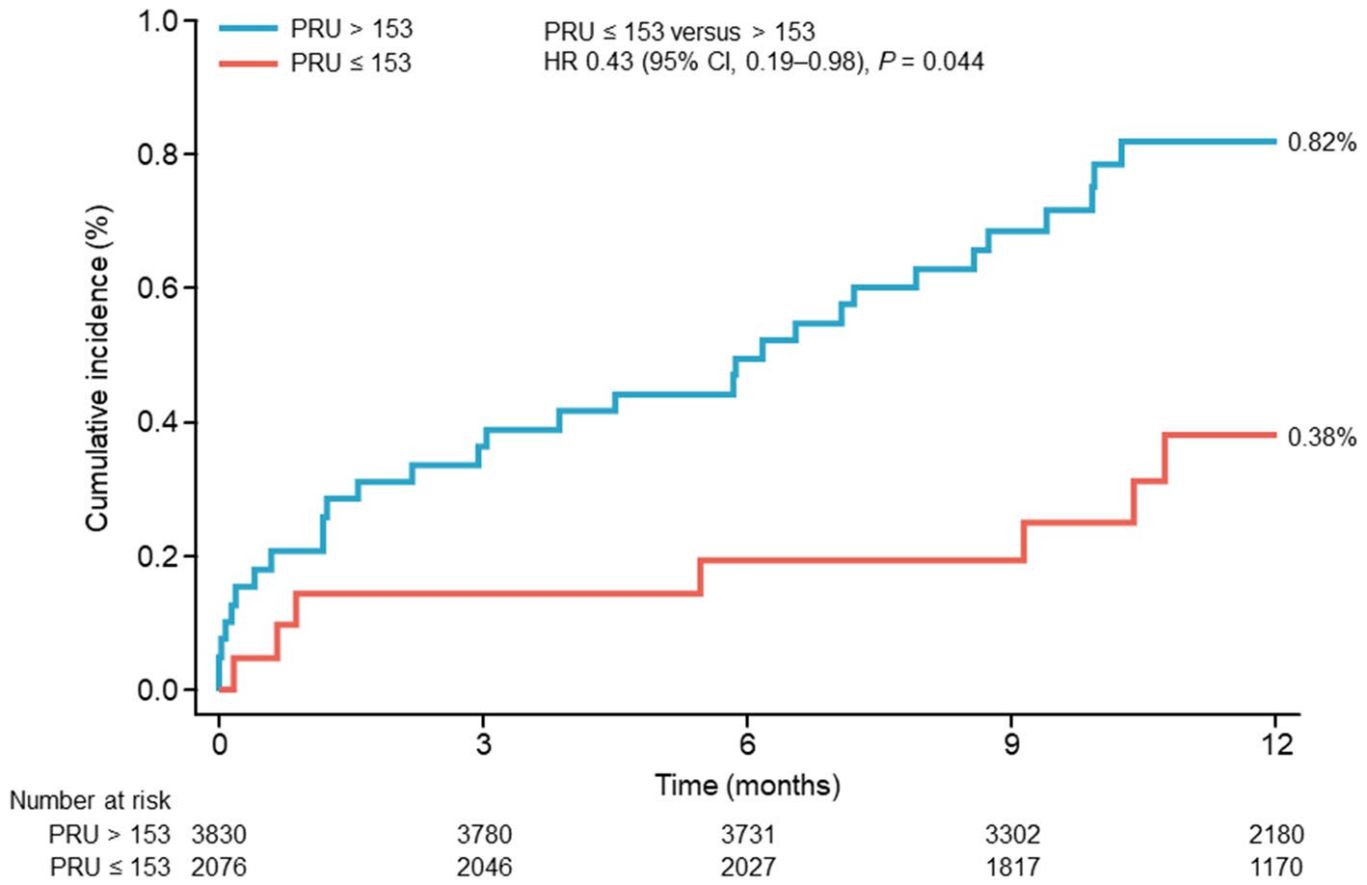
Cumulative incidence of non-fatal ischemic stroke at 12 months after the first PCI according to P2Y_12_ reaction unit value 12–48 h after the first PCI (≤ 153 versus > 153). *CI* confidence interval, *HR* hazard ratio, *PCI* percutaneous coronary intervention, *PRU* P2Y_12_ reaction unit

The area under the curve (AUC) and cutoff values of the ROC analysis for each outcome of this study are shown in Online Resource 2. As the representative values all had a low predictive ability, we focused on the CI; it was considered significant if the CI did not cross 0.5. Thus, the AUC of the ROC curve was significant at 0.601 (0.516–0.686) for non-fatal ischemic stroke but not at 0.508 (0.309–0.707) for non-fatal non-ischemic stroke. The cutoff for non-fatal ischemic stroke was a PRU of 153. When patients were stratified by PRU ≤ 153 versus > 153 at 12–48 h post-PCI, the cumulative incidence of non-fatal ischemic stroke at 12 months post-PCI was significantly associated with PRU (HR 0.43, 95% CI 0.19–0.98, *P* = 0.044; Fig. 4).

## Discussion

In this post hoc analysis of data from the prospective multicenter PENDULUM registry, we found that 47/5906 Japanese patients who underwent PCI and implantation of DES had a non-fatal stroke (37 ischemic strokes and 10 non-ischemic strokes) within 1 year after PCI. Additionally, post-PCI PRU levels were significantly higher among patients with a non-fatal ischemic stroke than those without a non-fatal ischemic stroke.

### Ischemic stroke risk in PCI patients

The information obtained in this study provides important implications from the perspective of primary and secondary prevention of ischemic stroke in patients undergoing PCI. In our analysis, 97.4% of Japanese patients undergoing PCI had one or more risk factors for ischemic stroke, indicating that patients undergoing PCI are also at high risk of ischemic stroke due to arteriosclerotic lesions.

The cumulative incidence of non-fatal ischemic stroke that we observed (0.68%) was lower than that for reported ischemic stroke in prior studies, such as PRASTRO-I (3.1–4.6%) [19] and CHANCE (10.2%) [20]. However, these studies did not differentiate between fatal and non-fatal stroke, as fatal ischemic stroke is generally a rare event; thus, these comparisons should be interpreted with care. Of note, those studies evaluated stroke recurrence rates in patients with ischemic stroke, suggesting that their study populations might be at a higher risk of ischemic stroke compared with the patients included in our study. In support of this, the current analysis found a higher incidence of ischemic stroke in patients with a history of ischemic stroke than in the overall population. This is in line with the STOPDAPT-2 randomized clinical trial, in which the rate of ischemic stroke at 1 year was 1.03% in patients who received 12 months of DAPT treatment [21].

Based on current Japanese guidelines, DAPT for preventing ischemic stroke recurrence should generally be restricted to a maximum of 3 months, with a short period of 3 weeks most commonly employed [3]. However, at the time of the PENDULUM study, it was recommended that DAPT be continued for at least 12 months to prevent stent thrombosis in PCI patients [3], and approximately 80% of PENDULUM patients continued DAPT treatment for 12 months. As a result, our data may reflect the outcomes of long-term DAPT in patients with ischemic heart disease. Our findings suggest that long-term continuation of DAPT may have benefits that override the adverse effects of DAPT among patients undergoing PCI.

Improved risk management for PCI patients may also have resulted in lower stroke rates in our analysis. While 82.8% of PENDULUM patients had a history of hypertension at baseline [17], optimal medical treatment was adopted. In addition, there were high prescription rates of dyslipidemia drugs and antidiabetic drugs, further controlling potential stroke risks.

### Association between non-fatal ischemic stroke and PRU after PCI

Although PRU values at 12–48 h post-PCI do not always match the PRU at the time of onset of ischemic stroke, many patients continued to use antiplatelet drugs at 12 months in this study. Most patients with non-fatal ischemic stroke events were taking P2Y_12_ inhibitors at the time of the event. Thus, we considered that PRU at 12–48 h after PCI was an appropriate marker to reflect the platelet aggregation ability at the time of ischemic stroke for patients in the PENDULUM registry.

The observation that patients with ischemic stroke events had significantly higher mean PRUs than those without events suggests that high PRU levels may be a risk factor for ischemic stroke. The trend towards a higher 1-year cumulative incidence in patients with HPR and a lower incidence in those with LPR supports this hypothesis. However, the differences between groups were not statistically significantly different, likely due to insufficient power because of the small numbers of events. In addition, the results of our previous report, in which the cardiovascular composite endpoint was an independent risk factor after adjusting for PRU, may also support the association between ischemic risk and PRU [17], as do the findings of a meta-analysis in which HPR at the time of P2Y_12_ inhibitor administration was shown to increase ischemic risk [11].

To date, evidence are lacking to suggest an association between HPR and stroke, and no cutoffs have been reported. In the open-label PRINCE trial of ticagrelor plus aspirin versus clopidogrel plus aspirin, HPR was associated with clinical outcomes for patients [22]. In addition, the PRINCE trial reported that the administration of clopidogrel significantly increased the risk of subsequent stroke compared with ticagrelor in patients who had an atherothrombotic stroke; furthermore, non-cardiogenic stroke, especially atherothrombotic ischemic stroke, tended to have a higher event incidence in patients with HPR compared with LPR [22]. There is no established cutoff PRU value for preventing ischemic stroke in patients undergoing PCI. Thus, we used the prespecified cutoff value (PRU > 208) to prevent cardiovascular events in patients undergoing PCI applied in the ADAPT-DES study [14] as reference. In this study, the calculated PRU cutoff for the onset of non-fatal ischemic stroke was 153; this is lower than the optimum PRU cutoff value of 206 for definite-probable and definite stent thrombosis calculated by ROC analysis in the ADAPT-DES study [14]. Although the discriminatory power for any event was poor or moderate, we can hypothesize that lowering the platelet aggregation ability is important for preventing ischemic stroke after PCI. However, this hypothesis requires further confirmation. Although the PRU cutoff value for the onset of non-fatal ischemic stroke was 153, the risk of non-fatal ischemic stroke was 0.43, which is consistent with a previous report [11].

### Non-fatal non-ischemic stroke

It is known that East Asians, including Japanese individuals, are more prone to cerebral hemorrhage than other ethnicities [23]. However, the cumulative incidence of non-fatal non-ischemic stroke (0.18%) in this analysis was low.

In the main PENDULUM analysis, few Japanese patients had excessively reduced platelet aggregation (LPR) while receiving P2Y_12_ treatment [17]. Therefore, although an inverse association between bleeding and PRU was reported in the ADAPT-DES study [14], no such relationship was observed in the PENDULUM population.

There are several possible reasons why PRU levels in Japanese patients may be reduced to a lesser extent than the European and US patients in the ADAPT-DES study. These include the impact of CYP2C19 polymorphism on clopidogrel [24, 25], and a higher proportion of poor metabolizers and a lower proportion of ultra-rapid metabolizers in Japan compared with other countries [26, 27]. Moreover, prasugrel is less susceptible to CYP2C19 variation, and, as a result, its efficacy is more stable across patient populations [28]. Of note, in Japan, approximately one-third of prasugrel prescriptions consist of low-dose prescriptions, meaning that the PRU levels in patients receiving low-dose prasugrel are reduced to the same extent as the levels observed in clopidogrel extensive metabolizers [29]. These hypotheses remain to be validated in future studies, and clinical analyses to evaluate the impact of long-term DAPT on the onset of a cerebral hemorrhage are warranted.

### Limitations

The main limitation of these data is the post hoc nature of the analysis; as the number of stroke events was small, statistical significance calculations were likely underpowered. Moreover, it is difficult to conduct some verifying analyses due to the small number of events. In addition, PRU was measured at 12–48 h after PCI and its level may have altered with medication changes during the observation period. However, because most of the patients who developed cerebral infarction continued DAPT, it is assumed that the PRU of patients who developed events was similar to that of 12–48 h after PCI. Nonetheless, the association between PRU and ischemic events observed in this analysis was consistent with the previously reported results of the PENDULUM registry [17]. We consider that these exploratory data provide important information for clinicians.

The exclusion of fatal strokes is another important limitation, meaning that data on severe strokes are still lacking. As most fatal strokes are due to cerebral hemorrhage [30], the results of this study may have underestimated outcomes related to ischemic stroke. However, as the proportion of fatal strokes in the PENDULUM study was low (data not shown), the influence of stroke mortality on the data reported herein is expected to be small.

In this study, the use of P2Y_12_ inhibitors was determined at the physician’s discretion, and the degree of platelet coagulation inhibition may vary depending on the type of drug administered. However, we consider that by evaluating PRU, an index of drug efficacy, we provided strong evidence for the links between platelet aggregation capability and stroke, regardless of the treatment regimen administered. Finally, the PENDULUM registry only included Japanese patients, which limits the generalizability of the findings. It is unclear whether our PRU cutoff is optimal for a secondary prevention trial of stroke.

## Conclusion

We have demonstrated that the cumulative stroke incidence 12 months post-PCI in Japanese patients was 0.68% for ischemic stroke and 0.18% for non-ischemic stroke, and that high PRU values at 12–48 h after PCI were associated with increased rates of ischemic stroke.

## Supplementary Information

Below is the link to the electronic supplementary material.

Supplementary file1 (PDF 98 KB)

Supplementary file2 (DOCX 27 KB)

## Data Availability

The deidentified participant data and the study protocol will be shared on a request basis for up to 36 months after the publication of this article. Researchers who make the request should include a methodologically sound proposal on how the data will be used; the proposal may be reviewed by the responsible personnel at Daiichi Sankyo Co., Ltd., and the data requestors will need to sign a data access agreement. Please directly contact the corresponding author to request data sharing. Once approved, the data will be shared in an appropriate way depending on the type of data requested.
