# Supplementary material for: Relationship between platelet aggregation and stroke risk after percutaneous coronary intervention: a PENDULUM analysis
Source: Heart Vessels. 2022 Jan 1;37(6):942–53. doi: 10.1007/s00380-021-02003-w (PMC9114031; doi:10.1007/s00380-021-02003-w)
Supplement: Supplementary file 1 — Supplementary file1 (PDF 98 KB) [file 380_2021_2003_MOESM1_ESM.pdf]

Electronic Supplementary Material 1

Relationship between platelet aggregation and stroke risk after percutaneous coronary intervention: a PENDULUM analysis

Heart and Vessels

Yuji Matsumaru, Takanari Kitazono, Kazushige Kadota, Koichi Nakao, Yoshihisa Nakagawa, Junya Shite, Hiroyoshi Yokoi, Ken Kozuma, Kengo Tanabe, Takashi Akasaka, Toshiro Shinke, Takafumi Ueno, Atsushi Hirayama, Shiro Uemura, Takeshi Kuroda, Atsushi Takita, Atsushi Harada, Raisuke Iijima, Yoshitaka Murakami, Shigeru Saito, Masato Nakamura

Corresponding author

Yuji Matsumaru

Division of Stroke Prevention and Treatment, Department of Neurosurgery, Faculty of Medicine, University of Tsukuba, Ibaraki, Japan

E-mail: yujimatsumaru@md.tsukuba.ac.jp

Online Resource 1. Antiplatelet status at first event

	All	Ischemic Stroke						Non-ischemic stroke
		All	Non-cardiogenic			Cardiogenic	Other	
			All	Atherothrombotic	Lacunar			
All	51	40	20	12	8	7	13	11
DAPT	43 (84.3)	36 (90.0)	20 (100.0)	12 (100.0)	8 (100.0)	6 (85.7)	10 (76.9)	7 (63.6)
Aspirin alone	5 (9.8)	3 (7.5)	0	0	0	1 (14.3)	2 (15.4)	2 (18.2)
$P2Y_{12}$ inhibitor alone	0	0	0	0	0	0	0	0

Data are *N* or *n* (%).

DAPT dual antiplatelet therapy.
