# Supplementary material for: Relationship between platelet aggregation and stroke risk after percutaneous coronary intervention: a PENDULUM analysis
Source: Heart Vessels. 2022 Jan 1;37(6):942–53. doi: 10.1007/s00380-021-02003-w (PMC9114031; doi:10.1007/s00380-021-02003-w)
Supplement: Supplementary file 2 — Supplementary file2 (DOCX 27 KB) [file 380_2021_2003_MOESM2_ESM.docx]

**Electronic Supplementary Material 2**

# Relationship between platelet aggregation and stroke risk after percutaneous coronary intervention: a PENDULUM analysis

*Heart and Vessels*

Yuji Matsumaru, Takanari Kitazono, Kazushige Kadota, Koichi Nakao, Yoshihisa Nakagawa, Junya Shite, Hiroyoshi Yokoi, Ken Kozuma, Kengo Tanabe, Takashi Akasaka, Toshiro Shinke, Takafumi Ueno, Atsushi Hirayama, Shiro Uemura, Takeshi Kuroda, Atsushi Takita, Atsushi Harada, Raisuke Iijima, Yoshitaka Murakami, Shigeru Saito, Masato Nakamura

## Corresponding author

Yuji Matsumaru

Division of Stroke Prevention and Treatment, Department of Neurosurgery, Faculty of Medicine, University of Tsukuba, Ibaraki, Japan

E-mail: yujimatsumaru@md.tsukuba.ac.jp

**Online Resource 2.** Receiver operating characteristic curve analysis of outcomes.

|  | **AUC** | **95% CI** | **Cut-off** |
| --- | --- | --- | --- |
| MACCE | 0.57 | 0.53–0.61 | 222 |
| All cause death | 0.55 | 0.50–0.60 | 221 |
| Non-fatal MI | 0.62 | 0.55–0.69 | 178 |
| Non-fatal stroke | 0.58 | 0.50–0.66 | 176 |
| Non-fatal ischemic stroke | 0.60 | 0.52–0.69 | 153 |
| Non-fatal non-ischemic stroke | 0.51 | 0.31–0.71 | NA |
| Stent thrombosis | 0.68 | 0.55–0.81 | 184 |
| Major bleeding | 0.53 | 0.48–0.58 | NA |

*AUC* area under the curve, *CI* confidence interval, *MACCE* major adverse cerebral cardiovascular event (composite of all cause death non-fatal MI non-fatal stroke and stent thrombosis), *MI* myocardial infarction, *NA* not available.
